# Development and validation of the prognostic value of ferritin in adult patients with Hemophagocytic Lymphohistiocytosis

**DOI:** 10.1186/s13023-020-1336-6

**Published:** 2020-03-12

**Authors:** Jun Zhou, Jing Zhou, Dan-Ting Shen, Hemant Goyal, Zhi-Qi Wu, Hua-Guo Xu

**Affiliations:** 1 Department of Laboratory Medicine, the First Affiliated Hospital of Nanjing Medical University, Nanjing, Jiangsu China; 2 Department of Internal Medicine, Mercer University School of Medicine, Macon, GA USA

**Keywords:** Hemophagocytic Lymphohistiocytosis, Adult, Prognosis, Ferritin, Overall survival

## Abstract

**Background:**

Hemophagocytic Lymphohistiocytosis (HLH) is a rare clinical syndrome with high mortality rate. The diagnosis of HLH draws on a constellation of clinical and laboratory abnormalities including extremely high serum ferritin levels. However, no biomarker has been firmly established as a clinically useful prognostic tool in HLH patients. We aimed to perform a retrospective analysis of two independent cohorts to explore the prognostic value of discharge serum ferritin for newly diagnosed adult HLH patients who recently started treatment. The prognostic value of serum ferritin levels at discharge (will be called as post-treatment ferritin level) was initially evaluated in a “test cohort” of 161 previously untreated consecutive adult HLH patients. It was then validated in a second cohort of 68 consecutive previously untreated patients (validation cohort).

**Results:**

Multivariate analysis revealed that significantly high post-treatment serum ferritin levels (>1050 μg/L) were associated with a higher risk of death and poor overall survival in the test cohort (hazard ratio (HR): 3.176, 95% confidence interval (CI) 1.468–6.869, *P* = 0.003), and the validation cohort (HR: 13.412, 95%CI 1.716–104.816, *P* = 0.013). At 6-month follow-up period in the test cohort, patients with a > 81% decrease in the serum ferritin level had a significantly higher probability of survival when compared with the patients with ≥14% increase in the serum ferritin level (94% vs. 31%, *P* < 0.001). Similar findings were observed on the analysis of the decrease in the serum ferritin level in the validation cohort.

**Conclusions:**

These results suggest that the serum ferritin level can be used as an independent prognostic marker in the adult HLH patients.

## Background

Hemophagocytic Lymphohistiocytosis (HLH) is a rare clinical syndrome [1, 2]. It is induced by an extreme immune activation of cytotoxic T-lymphocytes, natural killer (NK) cell and macrophages with an excessive cytokine production of tumor necrosis factor α (TNF-α) and interferon γ (IFN-γ), which is followed by hemophagocytosis [3–5]. HLH is broadly classified into primary HLH and secondary HLH according to the underlying etiologies [6]. Primary HLH occurs due to several genetic defects and is mainly found in children [4]. However, little is known about the adult HLH which can occur at any age and is correlated with a series of underlying conditions such as infectious diseases, malignancies and autoimmune diseases [7–9]. Although the incidence of HLH in adults is not precisely known, a Japanese observational study reported that up to 40% of HLH cases occur in adults [10, 11]. The mortality rate related to adult HLH can reach to 50–75% in the absence of appropriate treatment [12]. However, there are currently no satisfactory prognostic indicators to effectively assess the prognosis in adult patients with HLH.

Ferritin is an acute phase protein [13–15] which is found in various cells, tissues and organs of the body but especially prominent in macrophages, spleen and liver [16, 17]. Hyperferritinemia is very common in patients with HLH [18]. A very high serum ferritin level carries a better diagnostic sensitivity and specificity for HLH in the pediatric population [19, 20]. In a study by Lin et al. it was shown that a patient was 17 times more likely to die if decrease in ferritin was < 50% as compared to a 96% or greater decrease [21]. Prognosis of HLH in adults, in whom diagnostic and treatment approaches are more variable, is generally worse than in pediatric patients [22]. However, the relationship between serum ferritin and prognosis in adult HLH is unclear. Herein, we evaluate the utility of serum ferritin as a prognostic marker in two independent adult HLH cohorts.

## Results

### Characteristics of the test cohort

A total of 161 adult HLH patients (93 males, 68 females) met the included criteria for analysis. The initial diagnosis of adult HLH was made at median age 49 years (range, 18–88 years). The underlying etiologies HLH in these patients were mostly infectious diseases (*N* = 57, 35.4%), followed by malignancies (*N* = 39, 24.2%), and autoimmune disorders (*N* = 8, 4.9%), in 30 cases no underlying disorder was identified (Table 1). Multiple etiologies were found to be the cause of HLH in 27 cases (16.8%). Among patients in the test cohort, 96 patients received combination chemotherapy regimens or other cytotoxic drugs such as etoposide or cyclophosphamide; 65 of these patients received symptomatic treatment (methotrexate, steroids and supportive care). Baseline serum ferritin levels ranged from 188 to > 15,000 μg/L and 92.81% of patients had baseline serum ferritin level > 500 μg/L (the cutoff value of the diagnosis of HLH [9]) in the test cohort.

**Table 1:** The demographic, clinical and laboratorial characteristics of the patients included in the study

Characteristics	Test Cohort (***n*** = 161)					Validation Cohort (***n*** = 68)				All (***n*** = 229)		***P***-value^**∗**^
	IAHS (*n* = 57)	MAHS (*n* = 39)	MCTD (*n* = 8)	Mixed (*n* = 27)	Unclear (*n* = 30)	IAHS (*n* = 17)	MAHS (*n* = 14)	Mixed (*n* = 23)	Unclear (*n* = 14)	Test cohort (*n* = 161)	Validation cohort (*n* = 68)	
**General**												
**Gender (male/female), n**	37/20	24/15	3/5	16/11	13/17	10/7	9/5	14/9	5/9	93/68	38/30	0.793
**Median age (range), y**	52(18–88)	46(18–68)	44(19–59)	51(22–78)	44(18–74)	49(20–86)	59(21–76)	62(19–88)	54(47–78)	49(18–88)	55(19–88)	0.022
**Clinical features**												
**Fever,n**	53	37	7	25	30	16	13	22	13	152	64	1.000
**Mean T**_**max**_**,°C**	39.4	39.4	39.7	39.4	39.4	39.1	39.2	39.3	39.4	39.4	39.3	0.109
**Hepatomegaly**	8	9	0	6	9	2	0	2	4	32	8	0.140
**Splenomegaly**	14	15	0	10	12	2	6	4	5	51	17	0.312
**Lymph node enlargement**	8	11	1	8	7	1	2	4	2	35	9	0.136
**Rash**	5	4	4	2	10	3	1	2	1	25	7	0.297
**Jaundice**	13	5	1	5	7	3	1	2	4	31	10	0.412
**Edema**	10	5	3	5	6	5	2	6	4	29	17	0.228
**Bone marrow Hemophagocytosis**	41/45	33/37	5/7	17/26	24/26	14/14	11/12	19/19	10/12	120/141	54/57	0.060
**Laboratory data**												
**Ferritin ≥ 500 μg/L**	49/54	33/37	8/8	23/24	29/30	16/16	12/14	22/23	11/12	142/153	61/65	1.000
**EBV DNA copies (+)**	23/45	11/28	0/7	12/26	0/25	6/9	5/10	10/15	1/9	46/131	22/43	0.061
**FIB < 1.5 g/L**	15/51	11/33	1/6	4/24	3/27	4/14	4/13	3/20	2/8	34/141	13/55	0.944
**ANC < 1.0 × 10**^**9**^**/L**	12/56	9/36	0/8	7/25	2/29	3/17	5/14	7/23	4/13	30/154	19/67	0.144
**Hb < 90 g/L**	25/56	19/38	1/8	12/25	6/30	4/17	6/14	9/23	6/13	63/157	25/67	0.693
**Platelets** **<100 × 10**^**9**^**/L**	47/56	31/39	4/8	21/25	21/30	15/17	12/14	15/23	10/13	124/158	52/67	0.885
**ALT > 40 U/L**	43/56	29/38	7/8	13/25	21/29	10/16	7/14	17/23	9/13	113/156	43/66	0.278
**AST > 40 U/L**	44/56	30/38	7/8	18/25	22/28	12/16	11/14	17/23	9/13	121/155	49/66	0.537
**LDH > 271 U/L**	47/56	35/39	7/8	21/25	26/29	12/16	12/13	21/23	12/12	136/157	57/64	0.621
**DB > 6.8 μmol/L**	31/56	18/37	2/8	18/25	14/29	10/16	4/13	15/23	9/12	83/155	39/66	0.448
**TG > 3 mmol/L**	6/56	13/35	1/8	8/25	4/29	2/16	2/13	5/22	7/12	32/153	16/63	0.471
**Albumin < 40 g/L**	54/56	35/35	8/8	24/25	29/30	16/16	12/13	23/23	11/12	150/154	62/64	1.000
**Ca**^**2+**^ **< 2.20 mmol/L**	48/56	26/34	8/8	22/25	24/29	16/17	14/14	21/23	13/13	128/152	64/67	0.019

*ALT* Alanine aminotransferase, *ANC* Absolute neutrophil count, *AST* Aspartate aminotransferase, *Ca2+* Calcium, *DB* Direct bilirubin, *EBV* Epstein Barr virus, *FIB* Fibrinogen, *HB* Hemoglobin, *IAHS* Infection-associated HLH, *MAHS* Malignancy-associated HLH, *LDH* Lactate dehydrogenase, *MCTD* Mixed connective tissue disease, *Mixed* Mixed cause HLH, *TG* Triglyceride, *Unclear* Unknown underlying diseases; *Comparison with test cohort and validation cohort

In the test cohort, there was a positive correlation of post-treatment serum ferritin with alanine aminotransferase (ALT) (r_s_: 0.373, *P* < 0.001), with direct bilirubin (DB) (r_s_: 0.441, *P* < 0.001) and triglyceride (TG) (r_s_: 0.346, *P* < 0.001), and an inverse correlation with hemoglobin (HB) (rs: -0.258, *P* < 0.01), platelets (rs: -0.483, *P* < 0.001), with serum albumin (rs: -0.499, *P* < 0.001) and with serum calcium (Ca2+) (rs: -0.374, *P* < 0.001) (Supplemental Table 1).

### Post-treatment serum ferritin in the test cohort

**Fig. 1:**
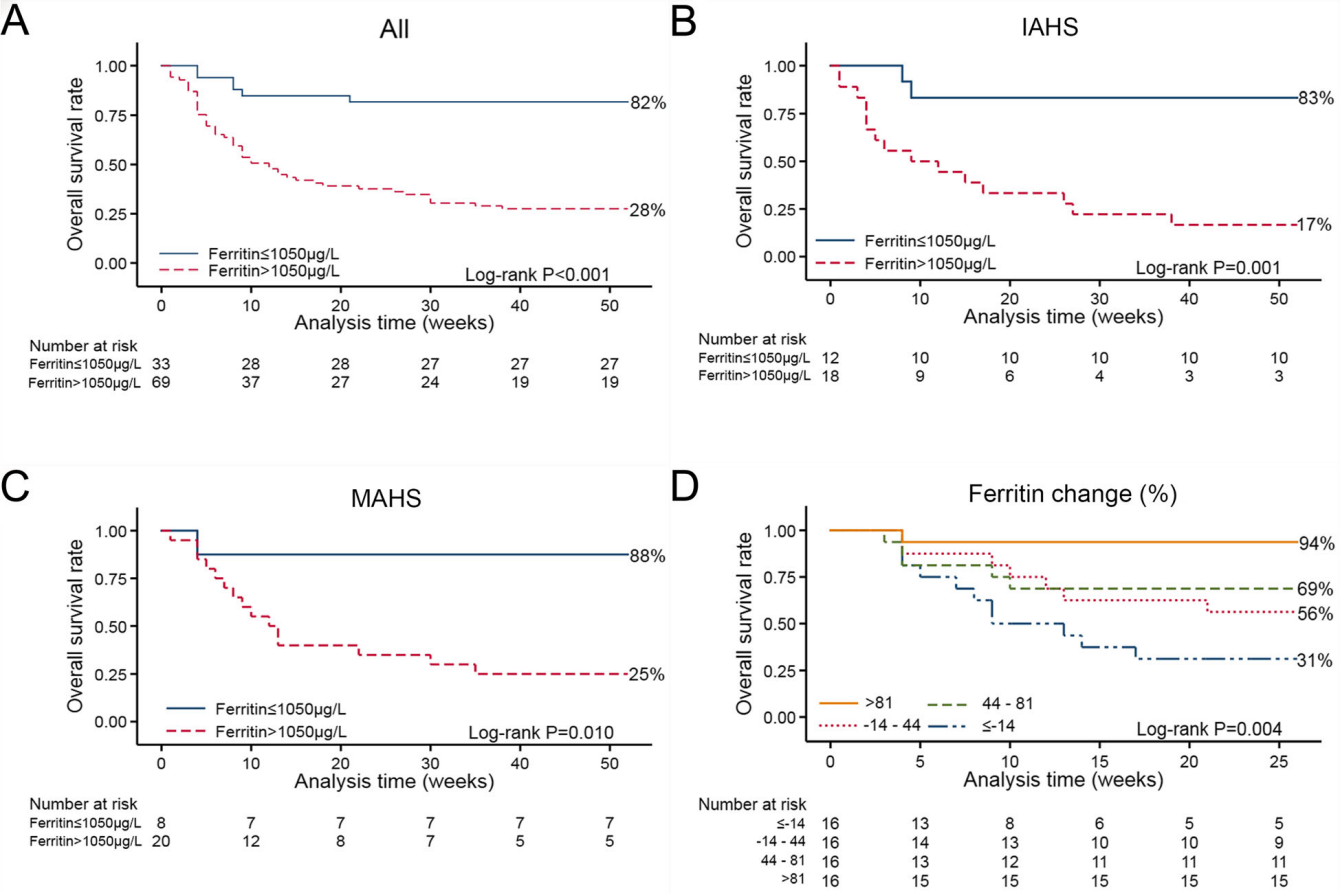
Performance of serum ferritin in different subgroups of adult HLH patients in the test cohort. **a** Adult HLH patients with post-treatment serum ferritin > 1050 μg/L showed significantly worse OS than those with post-treatment serum ferritin ≤1050 μg/L. **b** Infection-associated adult HLH patients with post-treatment serum ferritin > 1050 μg/L showed significantly worse OS than those with post-treatment serum ferritin ≤1050 μg/L. **c** Malignancy-associated adult HLH patients with post-treatment serum ferritin > 1050 μg/L showed significantly worse OS than those with post-treatment serum ferritin ≤1050 μg/L. (D) Overall survival, stratified according to quartiles of serum ferritin decrease

Median overall survival (OS) of the test cohort was 12 weeks, with a 1-year OS of 36.6%. Univariate analysis showed that an increased post-treatment serum ferritin levels were associated with lower survival (*P* < 0.001) (Supplemental Table 2). In order to identify an ideal cutoff level to evaluate the prognosis in the clinical practice, we performed a receiver operating characteristic (ROC) curve analysis with death at 1 year as a binary variable. Post-treatment serum ferritin level of > 1050 μg/L (area under the curve (AUC): 0.775, 95% CI 0.682–0.852, *P* < 0.001) (Supplemental Figure 1) was found to be a predictor of death at 1 year: 1-year OS was 28% and median OS was 12 weeks (vs 52 weeks for lower post-treatment serum ferritin) (Fig. 1a) (*P* < 0.001). We further conducted multivariate analyses with absolute neutrophil count (ANC), HB, platelets, ALT, DB, high density lipoprotein (HDL), albumin and Ca^2+^, all of which satisfied proportional hazards assumption (Supplemental Figure 2 and 3), and post-treatment serum ferritin continued to remain an independent prognostic marker for 1-year OS (hazard ratio (HR): 3.176, 95% confidence interval (CI) 1.468–6.869, *P* = 0.003) (Table 2).

**Table 2:** Multivariate analysis in the two cohorts. Using the cutoff values identified in the test cohort

Variables	Test Cohort			Validation Cohort		
	HR	95%CI	*P* - value	HR	95%CI	*P* - value
**Ferritin**	3.176	1.468–6.869	0.003	13.412	1.716–104.816	0.013
**ANC**	2.605	1.401–4.845	0.002	1.150	0.451–2.935	0.770
**Platelets**	2.314	1.208–4.431	0.011	3.612	0.772–16.901	0.103
**ALT**	2.459	1.365–4.430	0.003	0.714	0.277–1.841	0.486
**Ca**^**2+**^	2.061	1.030–4.124	0.041	3.223	0.411–25.300	0.266

*ALT* Alanine aminotransferase, *ANC* Absolute neutrophil count, *Ca*^*2+*^ Calcium, *HR* Hazard ratio, *CI* Confidence interval

To explore whether the above results also applied to different subgroups classified by underlying etiologies, we examined the performance of post-treatment serum ferritin level > 1050 μg/L within them (the relative data of HLH patients with autoimmune diseases were not evaluated due to small sample size). As shown by the Kaplan–Meier survival analysis patients with infection- or malignancy- associated HLH with serum ferritin > 1050 μg/L had poor OS (*P* = 0.001, *P* = 0.010, respectively) (Fig. 1b and c). In addition, 1-year OS with post-treatment serum ferritin levels > 1050 μg/L was 28% in the test cohort, 17% for infection-associated HLH patients and 25% for malignancy-associated HLH patients. The characteristics of the test cohort and different subgroups are summarized in Supplemental Table 3. There were no statistically significant differences among gender, median age, clinical features (fever, mean maximum temperature, hepatomegaly, splenomegaly, lymph node enlargement, rash, jaundice, edema, bone marrow hemophagocytosis) and laboratory data (ferritin, Epstein Barr virus (EBV) DNA, fibrinogen (FIB), ANC, HB, platelets, ALT, aspartate aminotransferase (AST), lactate dehydrogenase (LDH), DB, TG, albumin, Ca^2+^) between different subgroups within the test cohort, so we use the whole instead of subgroups to make further analysis.

### Post-treatment serum ferritin in the validation cohort

The demographic, clinical and laboratorial characteristics of patients (*N* = 68) in the validation cohort are summarized in Table 1. There were no statistically significant differences among age, clinical features and laboratory data evaluated earlier except for age (49 vs 55 years, *P* = 0.022) and Ca^2+^ (2.04 vs 1.96 mmol/L, *P* = 0.002) between the test and validation cohorts when patients were diagnosed initially.

**Fig. 2:**
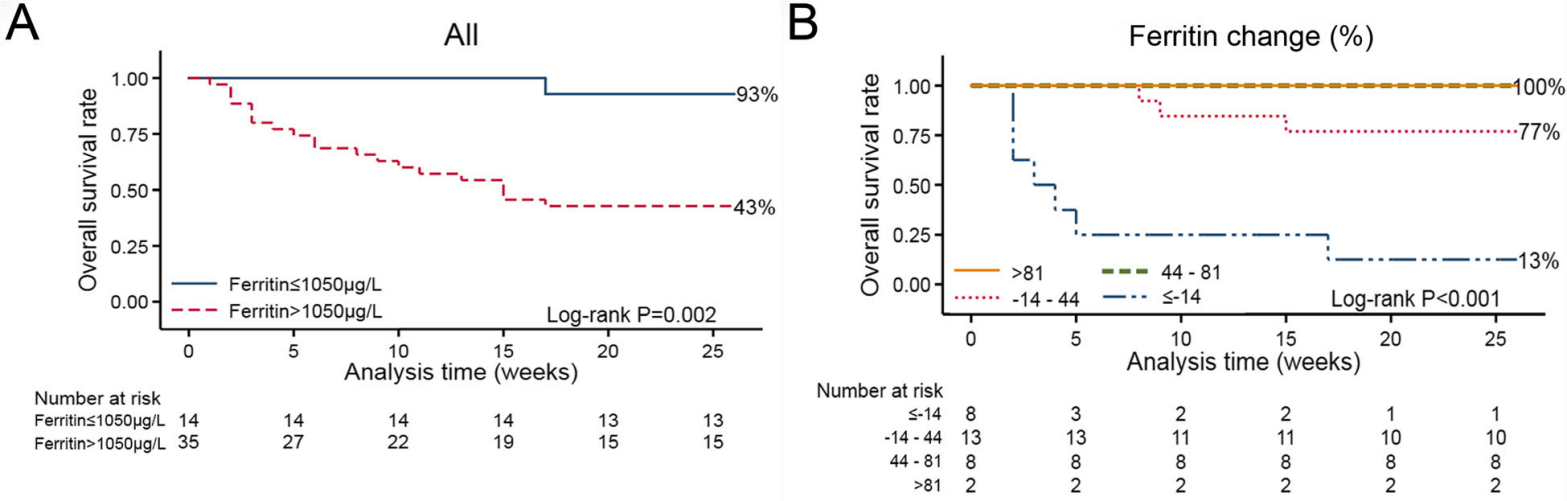
Performance of serum ferritin in the validation cohort. **a** Adult HLH patients with post-treatment serum ferritin > 1050 μg/L showed significantly worse OS than those with post-treatment serum ferritin ≤1050 μg/L. **b** Overall survival, stratified according to quartiles of serum ferritin decrease getting from the test cohort

We assessed whether the performance of post-treatment serum ferritin in the test cohort applies to the patients from the validation cohort. The median OS of the validation cohort was 26 weeks, with a 6-month OS of 55.9%. The 6-month survival of patients (post-treatment serum ferritin level ≤ 1050 μg/L) in the validation cohort was 93% vs 43% for patients those with higher levels (*P* = 0.002) (Fig. 2a). We carried out multivariate analysis by using the same variables and found that post-treatment serum ferritin was independently associated with survival (HR: 13.412, 95%CI 1.716–104.816, *p* = 0.013) (Table 2). We further investigated the prognostic value of post-treatment serum ferritin in the validation cohort with C-statistic. The C-statistic for post-treatment serum ferritin > 1050 μg/L was 0.708 (95%CI 0.561–0.829).

The results suggest that the post-treatment serum ferritin levels have independently prognostic significance for OS in two independent cohorts of adult HLH patients.

### Significance of serum ferritin decrease

**Fig. 3:**
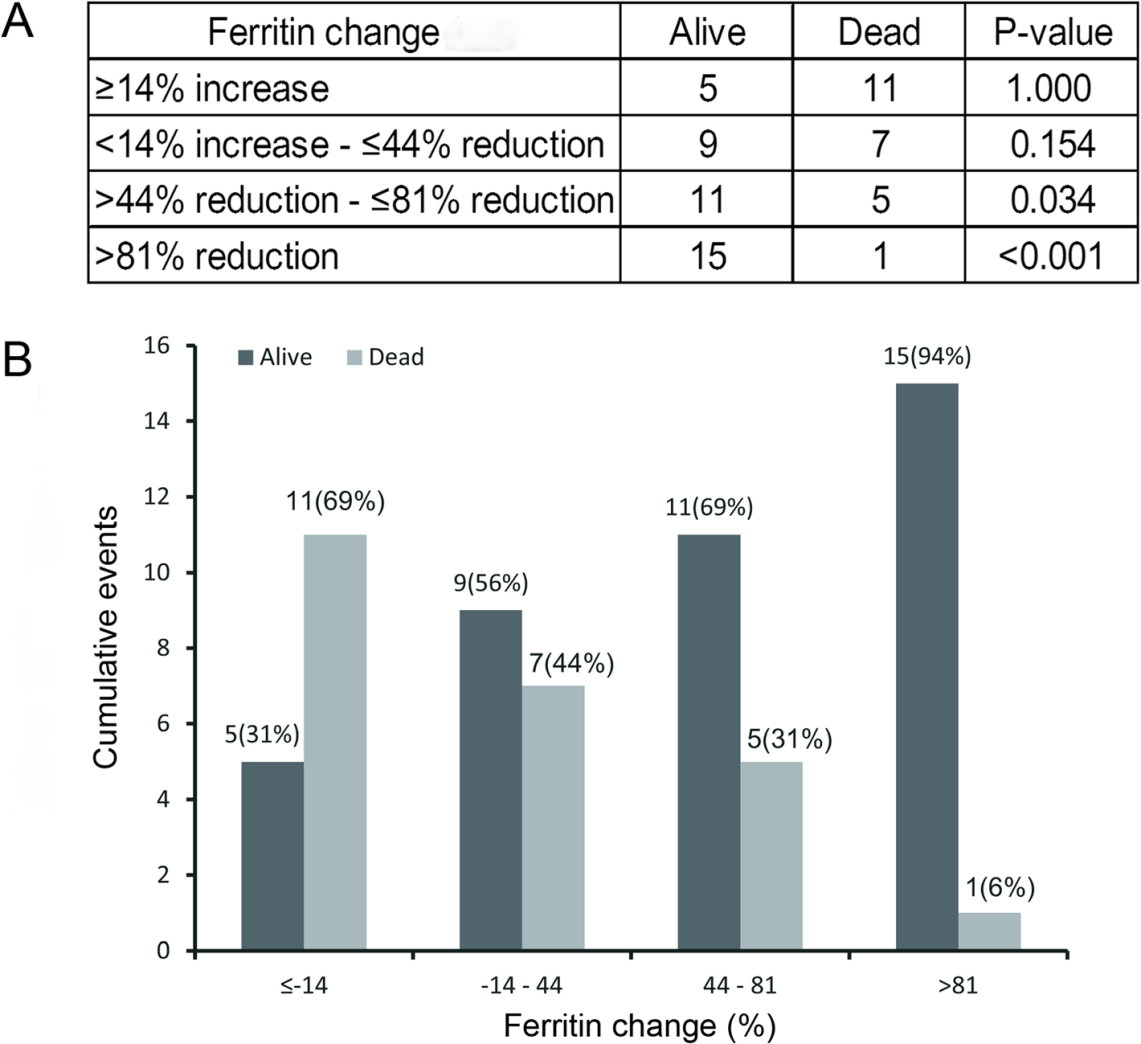
Adult HLH outcome, stratified according to quartiles of serum ferritin decrease in the test cohort. **a** Comparisons of adult HLH outcome between different quartiles. **b** The percentage of adult HLH outcome, stratified according to quartiles of serum ferritin decrease

The HLH patients with multiple serum ferritin measurements in the test cohort were further divided into four groups to determine the significance of decrease in the serum ferritin levels at 6 months after diagnosis. The results indicated that a decrease in the serum ferritin level was associated with OS. Of those patients having a > 81% decrease in serum ferritin levels: fifteen patients were alive and only one died. Of patients with ≥14% increase in serum ferritin only five patients were alive and eleven died at 6-month follow-up (6-month survival: 94% vs 31%, *P* < 0.001) (Fig. 1d and Fig. 3).

**Fig. 4:**
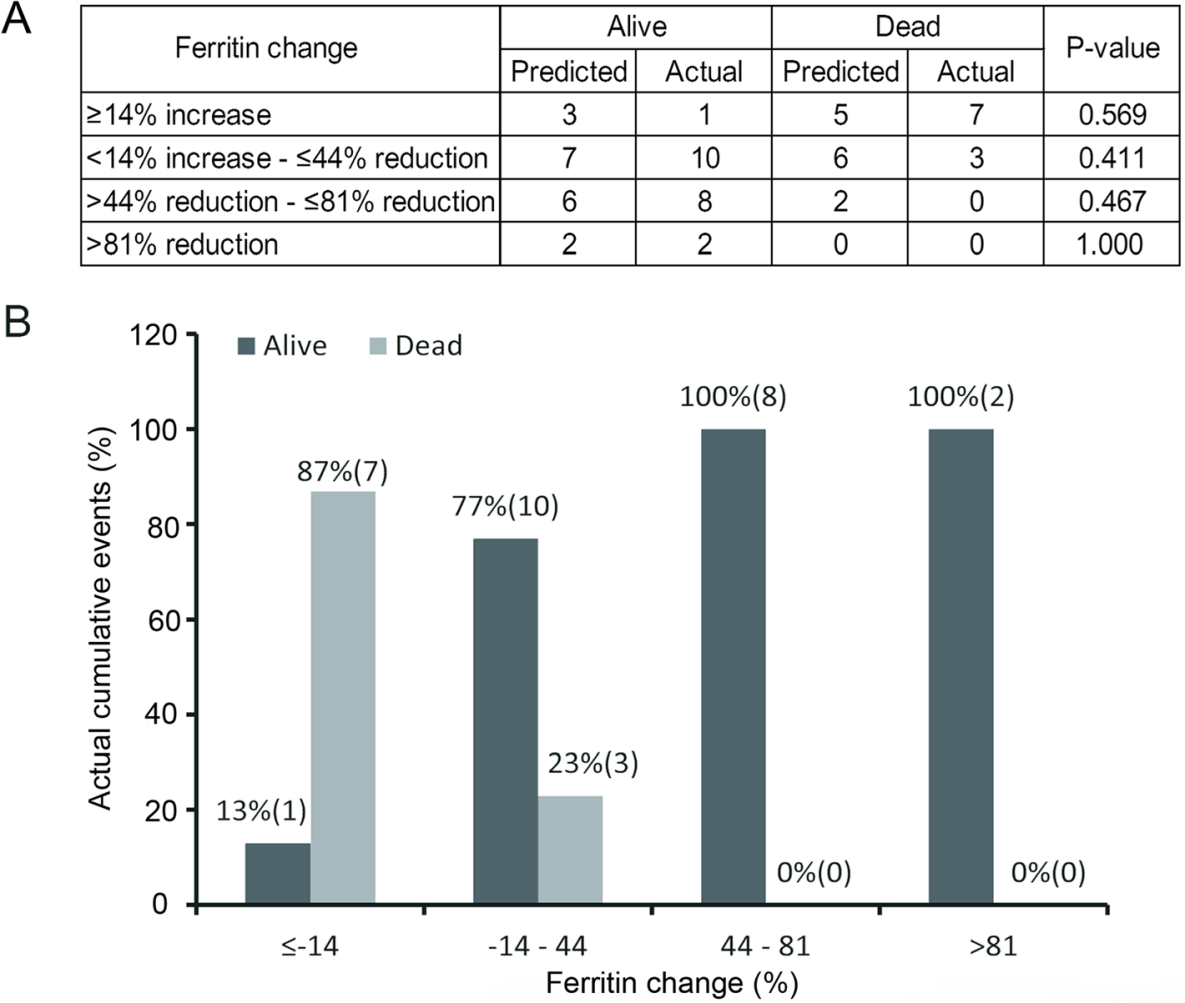
Adult HLH predictive outcome and actual outcome in the validation cohort. **a** Comparisons of adult HLH outcome between predictive outcome and actual outcome. **b** The percentage of actual adult HLH outcome, stratified according to quartiles of ferritin decrease

We further verified these findings in the validation cohort. A matrix of actual vs. predictive outcome using the decrease in the serum ferritin threshold identified in the test cohort is illustrated in Fig. 4. At 6-month follow-up, the predictive value: of patients with a > 81% decrease in serum ferritin levels, two were alive and no one died. Of those experiencing 44% decrease to 81% decrease in the serum ferritin, six patients were alive and two died. Of patients having 14% increase to 44% decrease, seven patients were alive and six died. Of patients with ≥14% increase in serum ferritin, three patients were alive and five died. The results show 77.4% of all patients being placed in correct categories in line with actual and there is no significant difference betweent the actual outcome and the predictive value (*P* = 1.000, *P* = 0.467, *P* = 0.411, *P* = 0.569, respectively) (Fig. 4). In addition, the 6-month survival of patients (serum ferritin decrease > 81%) in the validation cohort was 100 and 13% for those with serum ferritin increase ≥14%.

## Discussion

In the current study, we found that post-treatment serum ferritin level is an independent prognostic biomarker of adult HLH regardless of the underlying etiologies. Moreover, a greater decline in serum ferritin levels was associated with a better overall survival. Therefore, serum ferritin may be used to evaluate prognosis in adult HLH patients.

Most of the current research about serum ferritin are focused on its diagnostic abilities in HLH but not on its prognostic abilities. Although, hyperferritinemia has been reported to predict a poor prognostic outcome in adult malignancy-associated HLH [23, 24], these studies were inadequately powered, restricted to a single subgroup or a small population [25]. Moreover, the prognostic value of serum ferritin for other subgroups of adult HLH patients remains unclear. Thus, we conducted a retrospective observational study which is the largest study till date by using two independent cohorts and multiple HLH subgroups to improve the generalizability of the results. ROC analysis showed that post-treatment serum ferritin level greater than 1050 μg/L predicted mortality with a sensitivity of 89.3% in the test cohort which was better than a previous report [26] (serum ferritin > 2000 μg/L, sensitivity 71%). Kaplan–Meier survival analysis suggested that high post-treatment serum ferritin level (>1050 μg/L) can differentiate patients with poor OS from the test cohort, the infection- or malignancy-associated subgroups (*P* < 0.001, *P* = 0.001, *P* = 0.010, respectively). In the test cohort, univariate Cox proportional hazards regression analyses revealed that poor OS in adult HLH patients was associated with high post-treatment serum ferritin, ANC, HB, platelets, ALT, DB, HDL, albumin and Ca^2+^ level. Further, high post-treatment serum ferritin, ANC, platelets, ALT and Ca^2+^ level also found to be independent prognostic markers for OS in the multivariate analysis.

In the validation cohort, univariate analyses reflected that the poor OS was associated with high post-treatment serum ferritin level and platelets. Further, multivariate analysis revealed that high post-treatment serum ferritin level but not platelets was an independent prognostic marker in HLH. These results show that the post-treatment serum ferritin is more reliable for predicting prognosis than other variables in different cohorts. Wade et al. reported that it is simple models (incorporating few factors) rather than complex models (incorporating multiple factors) will be more likely to be similarly predictive in another cohort, for some of the included factors may be highly specific to one cohort rather than another cohort elsewhere [27]. This hypothesis appear to apply with our results. We applied post-treatment ferritin levels to two different cohort while ANC, platelets, ALT and Ca^2+^ were only suitable for one of them. There are also some factors could be underdiagnosed and under evaluated with the available data, but in this study from a practical viewpoint ferritin is more likely to be readily incorporated into clinical practice with minimal disruption.

In our cohort, serum ferritin decrease was analyzed as a categorical variable. Patients with > 81% serum ferritin level decrease had a significantly higher probability of survival compared with patients with serum ferritin increase ≥14% in the test cohort (6-month survival, 94% vs 31%, *P* < 0.001). These findings were also validated in the validation cohort. We concluded a higher decline in the serum ferritin levels was associated with a better OS. In contrast, in a small cohort of adult HLH patients with serial ferritin measurements (*N* = 40), Otrock et al. observed a correlation between the percent decrease in the ferritin level and 30-day mortality (*P* = 0.097), however, on the multivariate analyses, the prognostic value of percent decrease in ferritin was statistically compromised by other factors [23]. This discrepancy could be due to the small sample size and differing observation time (95 patients vs. 40 patients, 6 months vs. 30 days, respectively).

There are some limitations to this study. Because of the retrospective nature of study, we had to contend with some degree of missing clinical and laboratory investigation data or patients without follow-up. Although the results concur with the predictive outcome, the results may be biased due to a relatively small number of patients and the possibility that patients received better treatment in the validation cohort. Therefore, multicenter prospective randomized clinical trials with larger sample size are needed to further verify our results. Individual differences between the HLH patients and the disease management differs widely between physicians; therefore, it was not possible to timely monitor serum ferritin, and the selected highest serum ferritin level might not represent the peak serum ferritin level. We believe that the acquisition of true ferritin peaks will further increase the prognostic value of decrease in serum ferritin level.

## Conclusions

Post-treatment serum ferritin level can serve as an independent prognostic biomarker associated with early death, poor OS regardless of the underlying etiology for the adult HLH patients.

## Methods

### Patients

This was a retrospective cohort study conducted in the first affiliated hospital of Nanjing Medical University. The first cohort named “the test cohort” included 161 HLH patients between March 2010 and July 2016, and another cohort named “the validation cohort” included 68 HLH patients between August 2016 and June 2018. We used the following search engines at our institution: 1) SLEMR database capturing cases with a discharge diagnosis of HLH and extracted the clinical data, 2) Medical image data center to retrieve the pathology reports with HLH diagnosis. 3) NeulisFTP to capture the laboratory data. The inclusion criteria were: patients admitted with a primary diagnosis of HLH based on the originally proposed clinical criteria in 2004 (patients were needed to meet 5 of the following 8 criteria: 1) fever; 2) splenomegaly; 3) two or more cell lineage affected (HB <90 g/L, platelets <100 × 10^9^/L, neutrophils <1.0 × 10^9^/L); 4) hypertriglyceridemia ≥3 mmol/L and/or FIB ≤1.5 g/L; 5) hemophagocytosis in the bone marrow, spleen, or lymph nodes; 6) low or absent NK cell activity; 7) ferritin ≥500 μg/L; 8) soluble interleukin-2 receptor levels ≥2400 U/mL) [9], and patients with age ≥ 18 years. Patients in whom ferritin measurement was not performed before discharge or with unknown outcome were excluded. Our study is in accordance with the Declaration of Helsinki and approved by the Ethics Committee of The First Affiliated Hospital of Nanjing Medical University (Nanjing, China) (Ethical approval No. 2019-SR-066).

ANC, HB and platelets were analyzed using a Sysmex XE 2100 analyzers (Sysmex, Japan). ALT, AST, LDH, DB, TG, HDL, albumin and Ca^2+^ levels were performed by an AU 5800 Clinical Chemistry analyzers (Beckman Coulter, America). FIB levels were estimated by CS5100 system (Sysmex, Japan). EBV DNA was performed on Cobas Z480 (Roche, Switzerland). Ferritin analysis was performed on Unicel DXI 800 (Beckman Coulter, America).

The serum ferritin level assessed nearest to the date of diagnosis of HLH was considered as the pretreatment serum ferritin level and the one closest to the discharge date was named as the post-treatment ferritin level. The highest value of serum ferritin during the hospitalization was considered as the peak serum ferritin level. The decrease in the serum ferritin was calculated with the formula: serum ferritin change = 100 × (the peak serum ferritin - the post-treatment serum ferritin) / the peak serum ferritin.

### Statistical analysis

The optimal serum ferritin level cutoffs were first identified in the test cohort and then validated in the validation cohort. The optimal cutoff levels were defined by using the ROC analysis with death at 1 year as a binary variable. Depending on the distribution of data, associations of characteristics with the serum ferritin level were also expressed using Pearson’s or Spearman’s correlation. For comparisons of differences among groups, the *χ*^2^-test (Fisher’s exact test when appropriate) and the ANOVA or Mann-Whitney test were used. Overall survival was calculated from initial diagnosis date during hospitalization until the date of last follow-up or the date of death. Overall survival were plotted by the Kaplan-Meier estimator. Multivariate analysis was conducted using Cox proportional hazards. All analyses were done using SPSS 21 (IBM SPSS Statistics for Windows, Version 21. Armonk, NY). *P*-value less than 0.05 were considered statistically significant. Data was insufficient to test the prognosis of soluble interleukin-2 receptor (CD25) levels, NK cell function, or presence or absence of genetic mutations associated with adult HLH patients in both cohorts.

## Supplementary information

**Additional file 1: Table S1.** Correlations coefficients (rs) of ferritin with other characteristics. **Table S2.** Univariate analysis in the two cohorts. Using the cutoff values identified in the test cohort. **Table S3.** Cohort comparison between different underline diseases in test cohort. **Figure S1.** Performance of post-treatment serum ferritin level of >1050 μg/L in the test cohort. **Figure S2.** Schoenfeld residuals (graphical assessment) test for –In (−In (survival probability)). **Figure S3.** Schoenfeld residuals (graphical assessment) test for survival probability.

## Data Availability

The datasets analysed during the current study are available from the corresponding author on reasonable request.

## Abbreviations

ALT: Alanine aminotransferase
ANC: Absolute neutrophil count
AST: Aspartate aminotransferase
AUC: Area under the curve
Ca^2+^: Calcium
CI: Confidence interval
DB: Direct bilirubin
EBV: Epstein barr virus
FIB: Fibrinogen
HB: Hemoglobin
HDL: High density lipoprotein
HLH: Hemophagocytic lymphohistiocytosis
HR: Hazard ratio
IFN-γ: Interferon-γ
LDH: Lactate dehydrogenase
NK: Natural killer
OS: Overall survival
ROC: receiver operating characteristic
TG: Triglyceride
TNF-α: Tumor necrosis factor-α
