# Supplementary material for: Development and validation of the prognostic value of ferritin in adult patients with Hemophagocytic Lymphohistiocytosis
Source: Orphanet J Rare Dis. 2020 Mar 12;15:71. doi: 10.1186/s13023-020-1336-6 (PMC7068953; doi:10.1186/s13023-020-1336-6)
Supplement: Supplementary file 1 — Additional file 1: Table S1. Correlations coefficients (rs) of ferritin with other characteristics. Table S2. Univariate analysis in the two cohorts. Using the cutoff values identified in the test cohort. Table S3. Cohort comparison between different underline diseases in test cohort. Figure S1. Performance of post-treatment serum ferritin level of >1050 μg/L in the test cohort. Figure S2. Schoenfeld residuals (graphical assessment) test for –In (−In (survival probability)). Figure S3. Schoenfeld residuals (graphical assessment) test for survival probability. [file 13023_2020_1336_MOESM1_ESM.doc]

**Table 1 Correlations coefficients (r_s_) of ferritin with other characteristics**

| **Characteristics** | **r_s_** | **P-value** |
| --- | --- | --- |
| **General** |  |  |
| **Gender** | -0.250 | 0.011 |
| **Age** | 0.145 | 0.146 |
| **Clinical features** |  |  |
| **Fever** | -0.153 | 0.125 |
| **T_max_** | 0.093 | 0.367 |
| **Hepatomegaly** | -0.042 | 0.678 |
| **Splenomegaly** | 0.094 | 0.345 |
| **Lymph node enlargement** | -0.040 | 0.690 |
| **Rash** | -0.037 | 0.711 |
| **Jaundice** | 0.074 | 0.461 |
| **Edema** | -0.068 | 0.495 |
| **Bone marrow hemophagocytosis** | -0.063 | 0.549 |
| **Laboratory data** |  |  |
| **FIB** | -0.298 | 0.014 |
| **ANC** | -0.100 | 0.322 |
| **Hb** | -0.258 | 0.010 |
| **PLT** | -0.483 | <0.001 |
| **ALT** | 0.373 | <0.001 |
| **AST** | 0.583 | <0.001 |
| **LDH** | 0.508 | <0.001 |
| **DB** | 0.441 | <0.001 |
| **TG** | 0.346 | <0.001 |
| **ALB** | -0.499 | <0.001 |
| **Ca^2+^** | -0.374 | <0.001 |

**Table 2 Univariate analysis in the two cohorts. Using the cutoff values identified in the test cohort.**

| **Variables** | **Test cohort** | | | **Validation cohort** | | |
| --- | --- | --- | --- | --- | --- | --- |
|  | Hazard Ratio | 95%CI for HR | P- value | Hazard Ratio | 95%CI for HR | P- value |
| **Ferritin** | 4.602 | 2.175-9.739 | <0.001 | 11.623 | 1.555-86.855 | 0.017 |
| **ANC** | 2.332 | 1.369-3.971 | 0.002 | 2.160 | 0.878-5.310 | 0.093 |
| **Hb** | 2.566 | 1.575-4.182 | <0.001 | 1.947 | 0.831-4.563 | 0.125 |
| **PLT** | 2.932 | 1.623-5.297 | <0.001 | 3.716 | 1.098-12.575 | 0.035 |
| **ALT** | 2.000 | 1.176-3.400 | 0.011 | 1.630 | 0.683-3.890 | 0.271 |
| **DB** | 2.729 | 1.621-4.596 | <0.001 | 2.018 | 0.781-5.213 | 0.147 |
| **HDL** | 2.335 | 1.344-4.058 | 0.003 | 2.230 | - | 1.000 |
| **ALB** | 4.468 | 1.091-18.294 | 0.037 | 1.000 | - | - |
| **Ca^2+^** | 3.158 | 1.603-6.221 | 0.001 | 5.934 | 0.797-44.155 | 0.082 |

**Table 3 Cohort comparison between different underline diseases in test cohort**

| **Characteristics Patients** | **All**  **(n=161)** | **IAHS**  **(n=57)** | **P-value^∗^** | **MAHS**  **(n=39)** | **P-value^†^** | **Mixed**  **(n=27)** | **P-value^‡^** | **Unclear**  **(n=30)** | **P-value^§^** |
| --- | --- | --- | --- | --- | --- | --- | --- | --- | --- |
| **General** |  |  |  |  |  |  |  |  |  |
| **Gender (male/female), n** | 93/68 | 37/20 | 0.345 | 24/15 | 0.668 | 16/11 | 0.884 | 13/17 | 0.144 |
| **Median age (range), y** | 49(18-88) | 52(18-88) | 0.194 | 46(18-68) | 0.276 | 51(22-78) | 0.631 | 44(18-74) | 0.545 |
| **Clinical features** |  |  |  |  |  |  |  |  |  |
| **Fever ,n** | 152 | 53 | 0.948 | 37 | 1.000 | 25 | 1.000 | 30 | 0.391 |
| **Mean T_max_,℃** | 39.42 | 39.39 | 0.779 | 39.42 | 0.998 | 39.42 | 0996 | 39.44 | 0.859 |
| **Hepatomegaly** | 32 | 8 | 0.328 | 9 | 0.657 | 6 | 0.779 | 9 | 0.215 |
| **Splenomegaly** | 51 | 14 | 0.313 | 15 | 0.419 | 10 | 0.582 | 12 | 0.373 |
| **Lymph node enlargement** | 35 | 8 | 0.209 | 11 | 0.389 | 8 | 0.366 | 7 | 0.847 |
| **Rash** | 25 | 5 | 0.203 | 4 | 0.402 | 2 | 0.414 | 10 | 0.021 |
| **Jaundice** | 31 | 13 | 0.566 | 5 | 0.348 | 5 | 0.928 | 7 | 0.607 |
| **Edema** | 29 | 10 | 0.937 | 5 | 0.439 | 5 | 1.000 | 6 | 0.796 |
| **Bone marrow hemophagocytosis** | 120/141 | 41/45 | 0.304 | 33/37 | 0.525 | 17/26 | 0.016 | 24/26 | 0.503 |
| **Laboratory data** |  |  |  |  |  |  |  |  |  |
| **Ferritin≥500μg/L** | 142/153 | 49/54 | 0.847 | 33/37 | 0.694 | 23/24 | 0.912 | 29/30 | 0.435 |
| **EBV DNA copies (+)** | 46/131 | 23/45 | 0.058 | 11/28 | 0.676 | 12/26 | 0.287 | 0/25 | <0.001 |
| **FIB <1.5 g/L** | 34/141 | 15/51 | 0.457 | 11/33 | 0.276 | 4/24 | 0.423 | 3/27 | 0.135 |
| **ANC <1.0x10^9^/L** | 30/154 | 12/56 | 0.755 | 9/36 | 0.460 | 7/25 | 0.329 | 2/29 | 0.102 |
| **Hb <90 g/L** | 63/157 | 25/56 | 0.556 | 19/38 | 0.269 | 12/25 | 0.458 | 6/30 | 0.036 |
| **PLT <100x10^9^/L** | 124/158 | 47/56 | 0.382 | 31/39 | 0.891 | 21/25 | 0.527 | 21/30 | 0.311 |
| **ALT >40 U/L** | 113/156 | 43/56 | 0.527 | 29/38 | 0.628 | 13/25 | 0.039 | 21/29 | 0.998 |
| **AST >40 U/L** | 121/155 | 44/56 | 0.937 | 30/38 | 0.906 | 18/25 | 0.502 | 22/28 | 0.952 |
| **LDH >271 U/L** | 136/157 | 47/56 | 0.619 | 35/39 | 0.601 | 21/25 | 0.967 | 26/29 | 0.884 |
| **DBIL >6.8μmol/L** | 83/155 | 31/56 | 0.816 | 18/37 | 0.592 | 18/25 | 0.084 | 14/29 | 0.602 |
| **TG >3mmol/L** | 32/153 | 6/56 | 0.090 | 13/35 | 0.042 | 8/25 | 0.218 | 4/29 | 0.377 |
| **ALB <40 g/L** | 150/154 | 54/56 | 0.658 | 35/35 | 1.000 | 24/25 | 0.533 | 29/30 | 1.000 |
| **Ca^2+^ <2.20mmol/L** | 128/152 | 48/56 | 0.790 | 26/34 | 0.280 | 22/25 | 0.851 | 24/29 | 1.000 |

ALT, alanine aminotransferase; AST, aspartate aminotransferase; EBV, Epstein Barr virus; FIB, fibrinogen; IAHS, Infection-associated HLH; MAHS, Malignancy-associated HLH; LDH, lactate dehydrogenase; MCTD, Mixed connective tissue disease; Mixed, mixed cause HLH; TG, triglyceride; Unclear, unknown underlying diseases; ^∗^ Comparison with all and IAHS patients; ^†^ Comparison with all and MAHS patients; ^‡^ Comparison with all and mixed cause HLH patients; ^§^Comparison with all and unknown underlying diseases HLH patients.


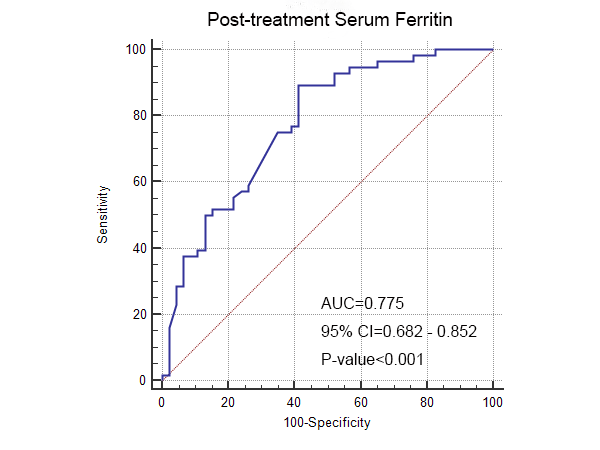


**Figure. 1** Performance of post-treatment serum ferritin level of >1050 μg/L in the test cohort


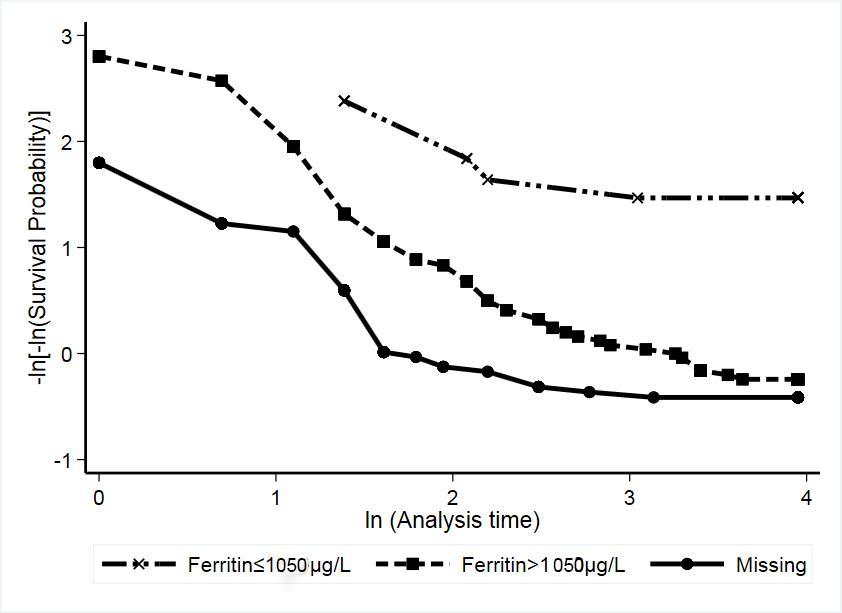


**Figure. 2** Schoenfeld residuals (graphical assessment) test for –In(-In(survival probability))


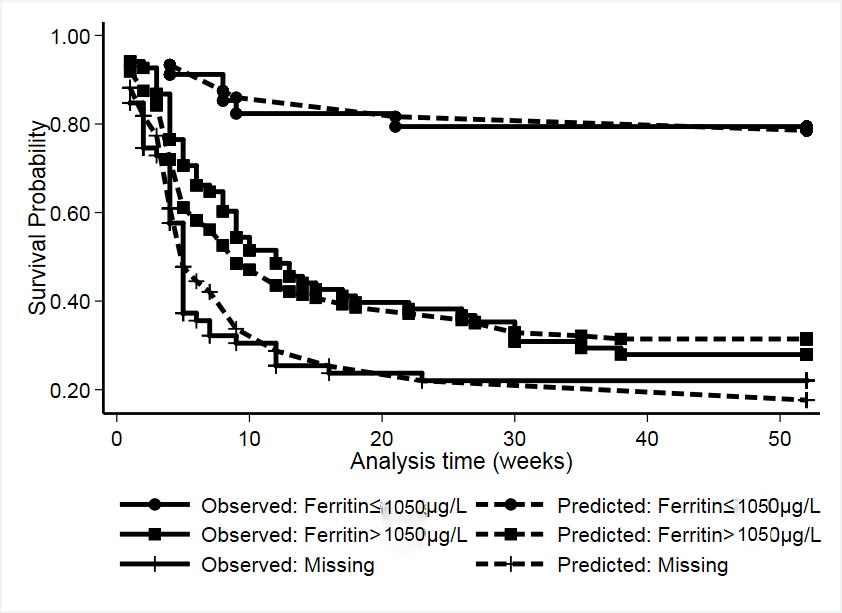


**Figure. 3** Schoenfeld residuals (graphical assessment) test for survival probability
